# Metabolic effects and cardiovascular disease risks of TDF or TAF in patients with chronic hepatitis B: a systematic review and meta-analysis

**DOI:** 10.3389/fphar.2025.1604972

**Published:** 2025-06-30

**Authors:** Yuan-Hai Zhou, Nan Cai, Yu-Xin Chen, Yong-Lu Su, Peng Hu

**Affiliations:** ^1^ Department of Infectious Diseases, The First Affiliated Hospital of Chongqing Medical University, Chongqing, China; ^2^ Institute for Viral Hepatitis, The Key Laboratory of Molecular Biology for Infectious Diseases, Chinese Ministry of Education, Chongqing Medical University, Chongqing, China; ^3^ Department of Infectious Diseases, The Second Affiliated Hospital of Chongqing Medical University, Chongqing, China; ^4^ Research Institute of Infectious Diseases and Parasitic Disease, Chongqing Key Laboratory of Infectious Diseases and Parasitic Disease, Chongqing, China

**Keywords:** dyslipidemia, metabolic profile, cardiovascular risk, tenofovir, drug safety, chronic hepatitis B

## Abstract

**Background and aims:**

The effects of Tenofovir Disoproxil Fumarate (TDF) or Tenofovir Alafenamide (TAF) on lipid profiles have been observed in chronic hepatitis B (CHB) treatment. However, the metabolic features and their impact on cardiovascular risk remain unclear. We conducted a systematic review and meta-analysis to evaluate these effects.

**Methods:**

We searched for studies from four major databases (PubMed, Web of Science, EMBASE, and the Cochrane Library) that reported the effects of TDF or TAF on metabolism and cardiovascular disease risk. The changes in metabolic parameters and 10-year atherosclerotic cardiovascular disease (ASCVD) risk were compared with baseline in the TDF and TAF treatment groups. Extracted data were analyzed with the random-effects model or the fixed-effects model. Potential sources of heterogeneity were investigated using sensitivity and subgroup analyses.

**Results:**

A total of 19 studies including 19,396 CHB patients (12,067 in TDF‐only group, 5,423 in TAF‐only group, and 1906 in TDF-switched group) were included in this meta-analysis. We found that both TAF and TDF treatment mildly increase the 10-year ASCVD risk. The TAF treatment showed significant increases in body weight, with no significant effects were observed on lipid levels or blood glucose. While TDF treatment has a lipid-lowering effect and caused weight loss. Subanalyses emphasized the impact of changing antiviral treatment strategies on metabolism. We found an increased risk of dyslipidemia and body weight gain after switching from TDF to TAF treatment.

**Conclusion:**

Although TAF and TDF treatments exhibit different metabolic characteristics, both mildly increase the risk of cardiovascular disease.

**Clinical Trial Registration:**

identifier CRD42024595452

## Introduction

Hepatitis B virus (HBV) remains a major public health concern, with an estimated 29.6 million infected individuals worldwide in 2019, causing considerable liver-related morbidity and mortality (Sh et al., 2022; Cui et al., 2023). Viral suppression can effectively improve the long-term prognosis of chronic hepatitis B patients, while also reducing the risk of progression to cirrhosis and hepatocellular carcinoma (Wei and Kao, 2017). Therefore, effective antiviral treatment is crucial for patients with chronic hepatitis B.

Tenofovir alafenamide (TAF) and tenofovir disoproxil fumarate (TDF), as first-line oral antiviral agents, are widely used and recommended by international guidelines (The Korean Association for the Study of the Liver KASL, 2022). However, to achieve the ideal treatment goal for chronic hepatitis B patients, which is functional cure, defined as HBsAg seroclearance or seroconversion (Anderson et al., 2021; Terrault et al., 2016). Most patients require lifelong treatment. Therefore, increased attention is being paid to the monitoring and management of comorbidities in the treatment of chronic hepatitis B under long-term antiviral treatment.

The previous studies have investigated whether the use of NA treatment increases the risk of potential complications. TAF was initially used to treat human immunodeficiency virus (HIV) infection before its efficacy was widely recognized (Hamers et al., 2013). However, in patients treated for HIV infection, TAF has been suspected of having potential metabolic side effects, such as elevated TC levels and an increased ASCVD risk (Huhn et al., 2020; Kauppinen et al., 2019). In contrast, TDF has been reported to have lipid-lowering effects in the treatment of HIV patients (Santos et al., 2015). Similarly, during the treatment for CHB, recent cohort studies have shown that TAF and TDF treatments are associated with changes in metabolic parameters (Zhang Q. et al., 2022; Shin et al., 2024). Pin-Nan Cheng et al. showed that the switch from TDF to TAF was associated with weight gain, derangements of lipid profile, and increased insulin resistance in patients with CHB (Cheng et al., 2024). However, dyslipidemia and metabolic syndrome (Mets) is a major risk factor for the development of ASCVD (Atar et al., 2021; Esposito et al., 2012). A recent prospective study reported that TAF had comparable risks of cardiovascular outcomes to those of patients treated with TDF (Fung et al., 2024).

Nevertheless, considerable debate persists in current research regarding whether metabolic parameter changes during NA treatment influence long-term cardiovascular risk in patients with chronic hepatitis B. Similarly, there is also no consensus regarding the effects of NAs on other metabolic parameters such as lipids, glucose, and body weight. Two recent systematic reviews focusing on the impact of NA treatment on lipid levels (Tong et al., 2024; Hwang et al., 2023), But they did not provide a comprehensive assessment of other metabolic parameters and cardiovascular disease risk. Additionally, prior systematic reviews did not include all randomized controlled trials of antiviral treatments.

The long-term cardiovascular outcomes and metabolic effects of antiviral therapies often require extended follow-up periods that are better captured by observational cohort studies. Hence, this meta-analysis incorporates both RCTs and observational studies to comprehensively assess the metabolic features and the risk of cardiovascular disease in patients with CHB under long-term treatment, in order to guide clinical decision-making and develop targeted treatment strategies for different patients.

## Methods

This protocol for systematic review and meta-analysis adheres to the Preferred Reporting Items for Systematic Review and Meta-Analysis Protocols (PRISMA) statement and is available on PROSPERO (CRD42024595452).

### Literature search

To identify all randomized controlled trials (RCTs) and cohort studies reporting on the treatment of chronic hepatitis B with TDF and TAF, we used the PICOS criteria as a guide for our search strategy. A comprehensive search was conducted across multiple databases, including PubMed, EMBASE, Cochrane Central Register of Controlled Trials, and Web of Science. We consulted with an experienced medical librarian to refine our search strategies for each database, aiming to identify all relevant studies published from the establishment of each database up to 1 September 2024. The detailed search strategy is provided in the Supplementary Material (Table 1). Additionally, we reviewed the reference lists of the included studies and relevant systematic reviews to identify other potential eligible studies.

**TABLE 1:** Characteristics of the studies showing the Metabolic effects and cardiovascular disease risks of TDF or TAF in patients with chronic hepatitis B.

Author, year	Study design	Number of patients	Treatment strategy	Main conclusion	NOS assessment
Cheng et al. (2024)	[PC]	TDF: 99 TAF: 99	Switch (TDF→TAF)	the ASCVD risk did not enhance in TDF-switched patients. The switch from TDF to TAF significantly increased body weight, triglyceride, total cholesterol, HDL, LDL, fasting glucose, insulin and insulin Resistance	8
Shin et al. (2024)	[RC-PSM]	TDF: 4150 TAF: 670	Mono and Switch	10‐years ASCVD risk was greater in the TAF‐only group than in the TDF‐only group. Both the TAF‐only and TDF‐only groups had a greater risk of developing ASCVD than the non‐antiviral group. Long‐term use of TAF increased total cholesterol, weight gain, and statin use	8
Fung et al. (2024)	[RCT]	TDF: 1632 TAF: 1632	Mono	the 10-year ASCVD risk was low and similar, with no significant differences in cardiovascular events, for both TDF and TAF treatment groups.TAF treatment period, the levels of LDL, TG and TC:HDL ratio significantly increased, whilst levels of HDL significantly decreased. Levels of fasting TC were unaffected	
Hong et al. (2023)	[RC-PSM]	TDF: 3186 TAF: 938	Mono	the risk of long-term cardiovascular events in treatment-naïve CHB patients treated with TAF and TDF were comparable and no significant difference in the TC/HDL ratio and levels of LDL Distinct serial changes between the two treatments were shown in lipid profiles	7
Zhao et al. (2024)	[PC]	TAF: 137	Mono	TC, TC/HDL and LDL levels gradually increased during treatment with TAF, TG levels gradually increased before 48 W and then gradually decreased. HDL levels did not change significantly during 96W of treatment with TAF.	8
Lai et al. (2023)	[RC-PSM]	TAF: 214	Mono	serum lipoprotein levels were considerably higher than the pretreatment levels for TC and TG, whereas HDL and LDL levels did not change in the TAF group	7
Byun et al. (2022)	[RCT]	TDF: 87 TAF: 87	Mono and Switch	body weight increased in the TAF group, whereas it decreased in the TDF group at week 48 from baseline. TC, LDL, and HDL levels at week 48 from baseline were significantly greater in the TAF group than in the TDF group, TC/HDL decreased slightly in both of the treatment groups	
Chen et al. (2022)	[RC]	TAF: 181 TDF: 158	Mono	the blood lipid levels in the TDF group were lower than those in the TAF group, especially the TC level, and the occurrence of hyperlipidemia was related to age, sex, and BMI.	8
Ogawa et al. (2022)	[RC]	TDF: 116 TAF: 116	Switch (TDF→TAF)	total, low-density lipoprotein high-density lipoprotein cholesterol and triglycerides were significantly increased after the switch to TAF. But the total to HDL cholesterol ratio remained unchanged	8
Lim et al. (2022)	[RC-PSM]	TDF: 2245 TAF: 502	Mono	the TDF group showed decreases in total cholesterol, triglyceride, HDL and LDL, whereas the TAF group showed various changes in these levels. But the changes in median LDL cholesterol from baseline did not significantly differ between the two groups	9
Guner et al. (2022)	[RC]	TDF: 427 TAF: 427	Switch (TDF→TAF)	Change in HDL, LDL and TC at baseline, 6th month and 12th month are not significant. Switching to TAF was associated with improved virologic and biochemical responses. Lipid parameters remained stable and the drug was well tolerated	9
Zhang Y. et al. (2022)	[PC]	TAF: 126	Mono	Tenofovir alafenamide treatment mainly affects the TC and TG level in patients with CHB. The LDL-C decreased slightly but not significantly	7
Suzuki et al. (2022)	[RC]	TDF: 69 TAF: 69	Switch (TDF→TAF)	Total cholesterol, HDL, LDL, and oxidized LDL levels increased significantly after switching to TAF.TDF was associated with significantly lower serum TC, HDL, and LDL levels	8
Karasahin et al. (2023)	[RC]	TDF: 110 TAF: 110	Switch (TDF→TAF)	TC, HDL, and TC/HDL ratio showed no significant differences between baseline and 3, 6, and 12 months in TDF-experienced or treatment-naive patients	9
Zhang Q. et al. (2022)	[RC-PSM]	TDF: 37	Mono	TDF, has a lipid‐ lowering effect in CHB patients and. The impact of TDF on cardiovascular events during long‐term treatment still needs further investigation	7
Yeh et al. (2022)	[RC-PSM]	TDF: 91 TAF: 91	Switch (TDF→TAF)	In real-world NUC-experienced CHB patients, unexpected body weight gain was observed after TAF switching	7
Jeong et al. (2022)	[RC-PSM]	TDF: 140 TAF: 70	Mono	In the TAF vs. non-HBV-infected control subjects, no between-group differences in TC, LDL, HDL, TC/HDL ratio, and LDL/HDL ratio were observed at 48 weeks, no significant change in TC was observed in the TAF group. TAF might not worsen the lipid profiles of subjects compared to non-HBV-infected controls and patients with inactive CHB.	9
Akdemir Kalkan et al. (2022)	[RC]	TDF: 237 TAF: 237	Switch (TDF→TAF)	low-density lipoproteins cholesterol was observed to be significantly higher after 6 months compared to baseline values And the TC increased significantly in the TAF group	8
Agarwal et al. (2018)	[RCT]	TDF: 432 TAF: 866	Mono	an elevated LDL level and fasting glucose were observed in patients receiving TAF. median changes in fasting lipid parameters were small in the TAF group while declines in all three fasting lipid parameters and triglycerides were observed in the TDF group	

Abbreviations: TAF, tenofovir alafenamide; TDF, tenofovir disoproxil fumarate; Mono, monotherapy (TDF/TAF, single-agent regimen); Switch (TDF→TAF), regimen change from TDF, to TAF; HDL, high-density lipoprotein; LDL, low-density lipoprotein; TC, total cholesterol; TG, triglyceride; ASCVD, atherosclerotic cardiovascular disease; PSM, propensity score matching; NOS, Newcastle-Ottawa Scale; RCT, randomized controlled trial; PC, prospective cohort study; RC-PSM, retrospective cohort study with propensity score matching; RC, retrospective cohort study.

### Eligibility criteria and study selection

In order to incorporate as many high-quality studies as possible, we selected the literature based on the following criteria. The inclusion criteria were as follows: (1) Clinical studies published in English; (2) Patients with chronic hepatitis B virus infection; (3) Randomized controlled trials and cohort studies using TAF or TDF as antiviral drugs. The exclusion criteria were as follows: (1) Full text not available; (2) Review articles or meta-analyses; (3) Case reports; (4) Studies with fewer than 10 patients; (5) Animal studies or experiments; (6) co-infected with HCV or HIV; (7) Articles with unavailable relevant data.

One researcher (Nan Cai) examined the titles and abstracts and removed obviously irrelevant reports. Two researchers (Yuanhai Zhou and Yuxin Chen) reviewed the remaining full-text reports to determine whether the studies met the inclusion criteria. Discrepancies were resolved through discussion or consultation with a third reviewer when necessary.

### Data extraction

Two authors (Z. Yuanhai and N. Cai) independently extracted data from eligible studies into a specially designed spreadsheet (Excel, version 16.0; Microsoft Corp), with any discrepancies resolved through discussion. The following data were extracted from each study: (1) Basic study information, including authors, publication year, study type, sample size, and follow-up duration; (2) Patient characteristics, including gender, age, and BMI; (3) Antiviral regimen; (4) All primary and secondary outcome measures; (5) description of the quality assessment. Outcome definitions are the changes in lipid profiles, blood glucose, body weight, and 10-year atherosclerotic cardiovascular disease (ASCVD) risk. The ASCVD risk score was selected as it has been widely adopted as the preferred CV risk stratification tool in chronic disease populations, including patients with liver diseases (Goff et al., 2014). All eligible studies utilized the 10-year ASCVD risk score as their primary analysis endpoint. This methodological consistency enabled standardized data extraction and valid meta-analysis across heterogeneous study designs.

### Quality assessment

Two reviewers (Z. Yuanhai and C. Yuxin) independently assessed the risk of bias in all eligible randomized controlled trials (RCTs) using the Cochrane Risk of Bias Tool (Higgins et al., 2011). We conducted quality assessments in 6 domains of this tool. Included the following domains: random sequence generation; allocation concealment; blinding of patients, healthcare practitioners, data collectors, outcome assessors, and data analysts; incomplete outcome data; selective reporting; and other sources of bias. If the review of all individual domains was considered to show a low risk of bias, the trials was assessed as having a low risk of bias. The trials with an uncertain risk of bias or high risk of bias in one or more individual domains were considered to have a high risk of bias. Studies judged to have a high risk of selection bias, performance bias or detection bias were excluded from the meta-analysis.

For eligible cohort studies, the same two reviewers conducted quality assessments based on the Newcastle-Ottawa Scale (NOS) (Ohri, 2025), evaluating 3 domains: selection bias, comparability bias and outcome bias. A NOS score greater than 7 was considered indicative of high-quality studies, and studies with a score below 4 were excluded as low-quality studies. Any disagreements were resolved through discussion or by consulting a third reviewer. The results of the quality assessment can be found in the Supplementary Table S3.

### Statistical analysis

Changes in lipid levels, blood glucose, weight and 10-year ASCVD score in the TAF and TDF treatment groups were expressed as mean differences with 95% confidence intervals. The χ2 test for heterogeneity provided an indication of between-trial heterogeneity. Additionally, to quantify statistical heterogeneity, we used the I^2^ statistic and the Cochrane Q test, which assess the proportion of variability between studies due to differences rather than chance. Significant heterogeneity was considered when I^2^ > 50% and p < 0.05, and a random-effects model was used. In the absence of significant heterogeneity, a fixed-effect model was applied.

Sensitivity analysis was conducted to validate the results and further test the robustness of our results. We decided *a priori* to perform subanalyses included age, sex, antiviral treatment strategies, follow-up duration and different regions. Given the limited number of randomized controlled trials (RCTs, n = 3), we conducted a pooled analysis incorporating both RCTs and observational studies. In accordance with the Cochrane Handbook for Systematic Reviews of Interventions (version 6.5) (Cochrane, 2025), subgroup analyses stratified by study design (RCTs vs. observational studies) were performed to minimize heterogeneity arising from combining distinct study types.

Publication bias was visually inspected using a funnel plot and We tested for funnel plot asymmetry with the Egger and Harbord tests. A p-value of less than 0.05 was considered statistically significant. All statistical analyses were performed using Stata version 14.0 (Stata Corp, College Station, TX, United States). Quality assessment of RCTs was conducted using Review Manager 5.3.

## Results

### Characteristics of included studies

**FIGURE 1:**
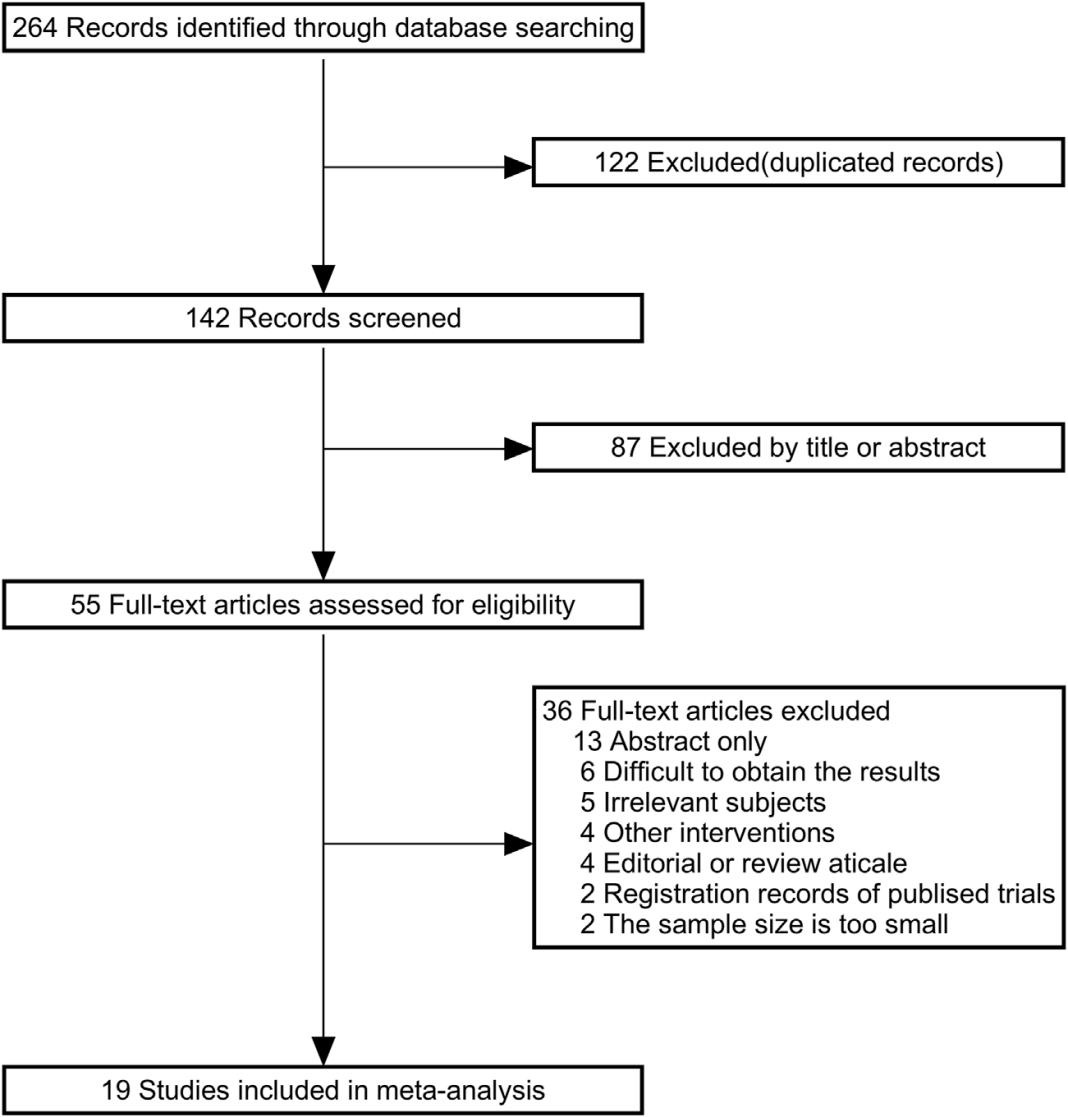
Flowchart for inclusion of studies in the meta-analysis.

Figure 1 shows the details of the study selection process. A total of 264 articles were identified through the initial bibliographic search strategy. After removing 122 duplicates and excluding 87 articles based on titles and abstracts, 55 studies were considered potentially relevant and underwent full-text review. Among these, 11 studies did not meet the inclusion or exclusion criteria. Of the remaining 44 studies, 13 only reported abstracts, 6 could not provide usable data, 4 were reviews, and 2 were clinical registry records. Finally, 19 studies were included. Information from the included studies is presented in Table 1.

The 19 studies included 19,396 chronic hepatitis B (CHB) patients (TDF group: 12,067 TAF group: 5,423, TDF-switched group: 1906). All studies were reported between 2018 and 2024, with a predominance of male participants. Most enrolled patients were between 40 and 60 years of age. Among the 19 studies, the most common design was retrospective cohort studies (13 studies), followed by 3 prospective cohort studies and 3 randomized controlled trials. 12 studies reported the effects of TAF and TDF on metabolism, while 9 studies showed the outcomes of switching from TDF to TAF. 6 studies discussed the impact of TAF and TDF treatment on cardiovascular disease risk in CHB patients. The quality assessment results of all eligible studies are shown in Supplementary Table S3.

### Changes in lipid levels with TAF and TDF

In the TAF group, we focused on analyzing lipid levels at 24, 48, 72 and 96 weeks, including TG, TC, HDL, LDL, and TC/HDL. The results showed that TAF treatment significantly increased TG and TC/HDL levels, while HDL decreased, with minimal effects on LDL and TC levels. During the follow-up period, the mean differences in TG from the baseline after 24, 48, 72 and 96 weeks of treatment were 3.69 mg/dL, 6.40 mg/dL, 6.97 mg/dL, and 9.25 mg/dL in the TAF group and 0.16 mg/dL,0.19 mg/dL, 0.20 mg/dL and 0.24 mg/dL in the TC/HDL ratio, respectively. In contrast, HDL levels showed a decreasing trend, at 24, 48, 72 and 96 weeks were −1.31 mg/dL, −1.72 mg/dL, −2.76 mg/dL and −3.98 mg/dL, respectively. TC levels showed a slight increase compared to baseline at 24 and 48 weeks, with increases of 3.52 mg/dL (95% CI, 0.91–6.12) and 1.83 mg/dL (95% CI, 0.05–3.51), respectively. However, after 72 weeks, there were no significant changes in TC levels. Similarly, LDL changes showed no significant differences after 48 weeks (Table 2). These findings were consistent across study designs in subgroup analyses, where both observational studies (TG: MD +7.39 mg/dL, 95% CI 3.12–11.65; HDL MD −1.03 mg/dL, 95% CI −1.32 to −0.73) and randomized controlled trials (RCTs) (TG MD +6.07 mg/dL, 95% CI 4.68–7.46; HDL MD −3.88 mg/dL, 95% CI −4.30 to −3.47) consistently demonstrated TG increases and HDL declines after 48-week TAF monotherapy (Supplementary Table S12).

**TABLE 2:** The changes of metabolic profile and ASCVD risk during TAF-only or TDF-only treatment (vs. baseline).

Variables of interest	TAF-only				TDF-only			
	N	MD (95% CI)	I^2^	p-value	N	MD (95% CI)	I^2^	p-value
Change in lipid profile								
TG (mg/dL)								
Week 24	4	3.69 (0.80, 6.57)	57.3%	0.07	5	−7.98 (-15.47, −0.45)	92.2%	0.01
Week 48	10	6.40 (3.60, 9.20)	80.0%	0.01	8	−6.41 (-10.68, −2.15)	93.5%	0.01
Week 72	4	6.97 (2.88, 11.06)	67.4%	0.02	4	−6.39 (-11.26, −1.53)	79.5%	0.02
Week 96	5	9.25 (7.79, 10.71)	46.4%	0.11	5	−5.19 (-10.06, −0.32)	85.5%	0.01
TC (mg/dL)								
Week 24	5	3.52 (0.91, 6.12)	77.2%	0.01	6	−12.70 (-18.47, −6.93)	95.1%	0.01
Week 48	11	1.83 (0.05, 3.51)	70.9%	0.01	10	−13.71 (-20.13, −7.29)	98.8%	0.01
Week 72	4	0.95 (-0.26, 2.16)	0.0%	0.44	3	−16.15 (-23.34, −8.95)	96.8%	0.01
Week 96	5	−0.21 (-1.25, 0.83)	0.0%	0.41	4	−18.21 (-25.44, −10.97)	97.6%	0.01
HDL (mg/dL)								
Week 24	4	−1.31 (-2.61, −0.02)	89.1%	0.01	4	−5.55 (-9.31, −1.79)	98.8%	0.01
Week 48	10	−1.72 (-3.04, −0.40)	93.2%	0.01	8	−5.49 (-9.45, −1.53)	99.1%	0.01
Week 72	4	−2.76 (-4.82, −0.70)	85.8%	0.01	3	−9.39 (-12.91, −5.88)	94.9%	0.01
Week 96	5	−3.98 (-5.35, −2.61)	85.4%	0.01	4	−10.58 (-12.73, −8.44)	90.7%	0.01
LDL (mg/dL)								
Week 24	4	2.59 (1.68, 3.49)	6.6%	0.36	4	−3.88 (-9.99,2.22)	93.9%	0.01
Week 48	11	2.04 (-0.72, 4.80)	89.9%	0.01	8	−4.54 (-8.36, −0.42)	95.9%	0.01
Week 72	4	2.22 (-2.48, 6.92)	87.4%	0.01	3	−7.44 (-11.27, −3.61)	70.9%	0.03
Week 96	5	1.65 (-2.48, 5.79)	91.7%	0.01	4	−8.78 (-11.64, −5.93)	72.2%	0.01
TC/HDL (mg/dL)								
Week 24	3	0.16 (0.11, 0.21)	55.6%	0.1	3	0.09 (0.06, 0.13)	48.1%	0.14
Week 48	5	0.19 (0.17, 0.21)	0.0%	0.68	4	0.11 (0.06, 0.17)	56.2%	0.07
Week 72	3	0.20 (0.10, 0.30)	74.6%	0.02	2	0.11 (0.01, 0.21)	71.8%	0.06
Week 96	4	0.24 (0.21, 0.26)	32.7%	0.21	3	0.16 (0.08, 0.24)	79.1%	0.01
Fasting glucose change (mg/dL)								
Week 48	2	2.14 (-1.86, 6.13)	86.7%	0.01	3	−1.67 (-4.67, 1.33)	58.1%	0.09
10-year ASCVD risk change (%)								
Week 48	3	0.67 (0.17, 1.17)	98.6%	0.01	2	0.55 (0.12, 0.98)	98.4%	0.01
Body weight Change (kg)								
Week 48	5	0.23 (0.13, 0.34)	7.6%	0.36	2	−0.54 (-0.64, −0.44)	0.0%	0.65

Abbreviations: TAF, tenofovir alafenamide; TDF, tenofovir disoproxil fumarate; HDL, high-density lipoprotein; LDL, low-density lipoprotein; TC, total cholesterol; TG, triglyceride; ASCVD, atherosclerotic cardiovascular disease; MD, mean difference; CI, confidence interval; N, the number of studies included in the meta-analysis.

The p value for heterogeneity is derived from the χ^2^ test and reflects the variation in effect sizes across studies.

^a^ 2 > 50% indicates significant heterogeneity.

In the TDF group, we observed a significant decrease in TG, TC, HDL and LDL levels throughout the entire follow-up period. At 48 weeks of treatment, the levels of TG, TC, HDL and LDL were −6.41 mg/dL (95% CI, −10.68 to −2.15), −13.71 mg/dL (95% CI, −20.13 to −7.29), −5.49 mg/dL (95% CI, −9.45 to −1.53), and −4.54 mg/dL (95% CI, −8.36 to −0.42), respectively (Table 2). Interestingly, similar to TAF, we also observed a slight increase in the TC/HDL ratio, which was +0.11 mg/dL (95% CI, 0.06–0.17) at 48 weeks. Importantly, this lipid-lowering profile of TDF remained consistent across study designs, with both RCTs and observational studies demonstrating concordant reductions in all lipid parameters (Supplementary Table S12).

### Changes in body weight and blood glucose with TAF and TDF

In view of the impact of TDF and TAF on blood lipids in CHB patients, it is important to explore whether they may also lead to other metabolic changes. Current research mainly focuses on whether TAF and TDF influence changes in body weight and blood glucose. We conducted a meta-analysis of existing studies, and the results showed that after 48 weeks of TAF treatment, the change in body weight compared to baseline was 0.23 kg (95% CI, 0.13–0.34). In the TDF treatment cohort, body weight slightly decreased (mean difference −0.54, 95% CI -0.64 to −0.44).

However, in our study, the effects of TAF and TDF on blood glucose were not significant. At 48 weeks, the changes in blood glucose were 2.14 mg/dL (95% CI, −1.86–6.13) in the TAF treatment group and −1.67 mg/dL (95% CI, −4.67 to 1.33) in the TDF treatment group (Table 2).

### Switch‐TAF group

Given the current concerns regarding the safety of switching from TDF to TAF treatment, this study included a total of 9 studies involving 1,906 patients, aiming to analyze the changes in blood lipids and body weight after switching to TAF treatment.

Compared to the baseline levels before switching, after 24 weeks of TAF treatment, serum TG, TC, HDL and LDL levels significantly increased, with changes of 8.48 mg/dL (95% CI, 1.72–15.24), 16.72 mg/dL (95% CI, 10.13–23.3), 4.17 mg/dL (95% CI, 2.19–6.15), and 11.13 mg/dL (95% CI, 7.81–14.45), respectively (Table 3). However, after switching to TAF treatment, the TC/HDL ratio did not show any increase (mean difference 0.04 mg/dL, 95% CI -0.02–0.1).

**TABLE 3:** The changes of lipid profile and weight after switched from TDF to TAF treatment 24 weeks.

Treatment strategy	Variables of interest	No. of studies	Mean difference	95% CI	I^2^	p-value
TDF→TAF	Change in lipid profile					
	TG (mg/dL)	5	8.48	1.72 to 15.24	55.3%	0.06
	TC (mg/dL)	6	16.72	10.13 to 23.3	81.9%	0.01
	HDL (mg/dL)	6	4.17	2.19 to 6.15	68.7%	0.01
	LDL (mg/dL)	6	11.13	7.81 to 14.45	59.1%	0.03
	TC/HDL (mg/dL)	4	0.04	−0.02 to 0.1	0.0%	0.09
	Body weight Change (kg)	3	0.73	0.23 to 1.2	0.0%	0.92

Abbreviations: TAF, tenofovir alafenamide; TDF, tenofovir disoproxil fumarate; HDL, high-density lipoprotein; LDL, low-density lipoprotein; TC, total cholesterol; TG, triglyceride; CI, confidence interval; N, the number of studies included in the meta-analysis.

The p value for heterogeneity is derived from the χ^2^ test and reflects the variation in effect sizes across studies.

I2>50% indicates significant heterogeneity.

Regarding the changes in body weight, the results showed that after 24 weeks of switching to TAF treatment, body weight significantly increased compared to baseline (MD, 0.73; 95% CI, 0.23–1.2).

### Changes in 10-year cardiovascular risk

To determine whether the impact of TAF and TDF on lipid profiles during long-term treatment of CHB patients could exacerbate the risk of cardiovascular diseases, we systematically reviewed the existing literature and identified 6 relevant studies. Of these, 3 studies including a total of 8,282 patients were included in a meta-analysis. After 48 weeks of treatment with TAF and TDF, the estimated 10-year ASCVD risk change for the TAF-only treatment group was 0.67 (95% CI, 0.17–1.17), while for the TDF-only treatment group, it was 0.55 (95% CI, 0.12–0.98). The results clearly indicate that both TAF and TDF treatments are associated with an increased risk of cardiovascular disease (Table 2).

## Discussion

To our knowledge, the present meta-analysis is the first study that clearly addresses the impact of NA drugs on host metabolism and cardiovascular disease risk during CHB treatment. A total of 16 cohort studies and 3 randomized controlled trials (RCTs), involving 19,396 CHB patients, were evaluated. The results indicate that both TAF and TDF treatments can slightly increase the risk of ASCVD. Except for being associated with weight gain, TAF does not have a significant effect on other metabolic parameters. However, TDF treatment is associated with reductions in weight and lipid levels. Interestingly, the subgroup analysis of patients switched to TAF treatment revealed a marked increase in lipid levels.

Previous studies have shown that HBV infection, unlike HCV infection, is negatively correlated with lipid metabolism and may therefore have a protective effect on Mets, hepatic steatosis, and cardiovascular diseases (Wong et al., 2012; Wang et al., 2020). However, numerous studies have found that NA treatment have different effects on metabolic functions during long-term antiviral treatment. The impact of TAF on lipid profiles remains controversial. A cohort study found that TAF increased the levels of TC, TG and LDL, with no significant effect on HDL (Zhao et al., 2024). Similarly, in a recent RCT involving 1,632 participants, it was found that TAF treatment increased LDL, TG and TC/HDL levels while decreasing HDL levels, with no impact on TC (Fung et al., 2024). Consistent with these findings, our meta-analysis confirmed that TAF treatment lowers HDL levels without markedly influencing TC levels.

However, A previous meta-analysis showed that, compared with other antiviral drugs, TAF worsened lipid profiles after 6 months of treatment (Hwang et al., 2023). The reason why their findings regarding changes in Serum lipid levels, especially HDL, are inconsistent with ours may be that the number of studies included in the previous meta-analysis of each lipoprotein type was limited. A recent network meta-analysis reported no significant difference in lipid levels between TAF-treated and untreated CHB patients (Tong et al., 2024). This finding is consistent with our results regarding the impact of TAF on lipid levels. But our systematic review is updated and more comprehensive, providing more accurate evidence. We assessed changes in lipid levels over time and further explored the impact on risk of cardiovascular disease and other metabolic characteristics.

For patients with high cardiovascular (CV) risk, the 2016 ESC/EAS Guidelines recommend achieving either an LDL level <1.8 mmol/L (70 mg/dL) or a ≥50% reduction from baseline when baseline LDL ranges between 1.8 and 3.5 mmol/L (70–135 mg/dL) (Catapano et al., 2016). However, while multiple studies—including our meta-analysis—demonstrate statistically significant metabolic effects of TDF (e.g., LDL reduction: −8.78 mg/dL [95% CI: −11.64 to −5.93]; weight reduction: −0.54 kg [95% CI: −0.64 to −0.44]), these modest changes may be insufficient to achieve the ESC/EAS-recommended treatment targets for high-risk patients. Therefore, we conclude that TDF’s impact on metabolic parameters, though statistically significant, remains clinically marginal for high-CV-risk populations.

Metabolic disorders are the main characteristic of metabolic syndrome (Mets), which is a clustering of obesity, dysglycemia, dyslipidaemia (Esposito et al., 2012). Thus, our meta-analysis primarily concentrated on the changes in these metabolic parameters. As in previous studies (Shin et al., 2024; Cheng et al., 2024; Yeh et al., 2022; Byun et al., 2022), our results indicate that TAF significantly increases body weight, while weight decreases with TDF treatment. Weight management has gained increasing clinical attention. Although our meta-analysis demonstrated a weight-reducing effect of TDF, the observed magnitude of change failed to reach the ideal target range specified in the 2013 AHA/ACC/TOS Guideline for the Management of Overweight and Obesity (Jensen et al., 2014), the mechanisms underlying the differential effects of TDF and TAF on body weight remain an important area of investigation, particularly given their implications for cardiovascular disease risk. Hill A et al. demonstrated that TDF is associated with a 5% weight loss risk in HIV-negative individuals receiving pre-exposure prophylaxis (PrEP) (Shah et al., 2021; Bosch et al., 2023), which mechanistically parallels the weight reduction effect (Δ = −0.54 kg) observed in HBV-infected patients receiving TDF in our study. This bidirectional phenomenon may originate from two distinct mechanisms. First, gastrointestinal toxicity could play a role, as TDF may induce nausea and malabsorption in non-infected populations through its prodrug metabolism pathway. Second, regarding adipogenic regulation, TAF appears to enhance adipocyte differentiation via PPARγ pathway activation in HIV-infected states, while TDF may partially counteract this effect through PPARγ suppression (Dulion et al., 2025).

The differential effects of TAF and TDF on metabolic profiles and estimated ASCVD risk have attracted considerable attention. Hyunjae Shin et al. showed that both TAF and TDF increased the risk of cardiovascular disease during long-term CHB treatment, with TAF having a more significant effect (Shin et al., 2024). However, an RCT from Canada and a large cohort study from South Korea found that the 10-year ASCVD risk in CHB patients treated with TAF and TDF was low and similar, with no significant difference in cardiovascular events (Fung et al., 2024; Hong et al., 2023). Pin-Nan Cheng et al. also found that the 10-year ASCVD risk did not increase after switching from TDF to TAF treatment (Cheng et al., 2024). To address the current controversy, we included six studies, three of which were included in the meta-analysis. Due to the lack of comparative data between the TAF and TDF groups, we did not analyze the differences in their effects on ASCVD risk. Our meta-analysis indicates that both TAF and TDF mildly increase the 10-year ASCVD risk. But the change remains within acceptable limits.

High LDL levels and low HDL levels are considered independent risk factors for cardiovascular disease (Assmann and Gotto, 2004), Recent studies have reported that the TC:HDL ratio has greater predictive value than individual parameters (Gimeno‐Orna et al., 2005; Calling et al., 2019), and an increased TC:HDL ratio is associated with a higher risk of cardiovascular disease (Manubolu et al., 2022). Although there is significant heterogeneity among the included studies, our results show that both TDF and TAF increase the TC/HDL ratio and decrease HDL levels, thereby supporting our conclusion that both TDF and TAF treatment may increase the ASCVD risk. The 2018 AHA/ACC Guideline on the Management of Blood Cholesterol classifies a 10-year ASCVD risk <5% as low-risk (Grundy et al., 2019). Based on our findings, we recommend regular monitoring of CVD risk factors (e.g., hypertension, dyslipidemia) in CHB patients during treatment to identify those at elevated CVD risk.

Our systematic review and meta-analysis also provide the latest evidence on the safety of the treatment regimen of switching from TDF to TAF, in our subanalyses, we found a sharp increase in blood lipid levels after the switch to TAF treatment. Although we did not perform a meta-analysis of cardiovascular disease risk in the subgroup analysis, current studies suggest that ASCVD risk does not change after switching to TAF treatment (Cheng et al., 2024). However, we found that LDL levels, which are considered a risk factor for cardiovascular disease (Assmann and Gotto, 2004), rise sharply after switching to TAF treatment. Therefore, for populations with CVD risk factors, such as smoking, obesity and dyslipidemia, switching to TAF treatment requires comprehensive evaluation and careful consideration.

Our study had several limitations. First, consistent with prior meta-analytic approaches, we incorporated both randomized controlled trials (RCTs) and non-randomized observational studies to comprehensively synthesize all available evidence. While this approach may introduce significant heterogeneity and potential publication bias across studies, we mitigated these limitations through four key methodological strategies: (1) application of a random-effects model to enhance the generalizability of inferences beyond the study populations, (2) pre-specified subgroup analyses stratified by study design (RCTs vs. observational studies) to explore sources of heterogeneity, (3) we conducted a rigorous methodological quality assessment of the included studies, and only cohort studies with a NOS score greater than 6 were included in this meta-analysis, and (4) sensitivity analyses to verify the robustness of pooled estimates. Second, although multiple algorithms exist for estimating 10-year cardiovascular risk, our meta-analysis specifically employed the Pooled Cohort Equations (PCE) model—derived from U.S. populations—to calculate ASCVD risk scores, as it currently represents the most validated tool, and all included studies adopted this method as their analytical endpoint. However, this approach may limit the generalizability of our findings to other ethnic groups or populations with substantially different demographic characteristics. Third, during the data collection phase, lipid and cholesterol data were presented using median and interquartile range in some studies due to the non-normal distribution of these variables. To avoid losing high-quality studies, we addressed this issue by extracting data using the sample mean and standard deviation estimation tools recommended by Luo et al. (2018). This may affect the precision of the results. Fourth, because of insufficient data, we were unable to perform the subgroup analysis as planned, and the effects of NA treatment on different genders and age groups have not been fully assessed. There is a strong need for a meta-analysis in the future when sufficient data become available.

In conclusion, long-term TAF treatment is associated with weight gain, but has no significant effect on blood lipids and blood glucose. In contrast, long-term TDF treatment is associated with lipid-lowering effects and weight loss. However, both TDF and TAF increase the TC/HDL ratio and decrease HDL levels, thereby mildly raising the ASCVD risk. Clinicians should consider the potential risks of metabolic disorders and CVD during long-term NA treatment, especially when switching to TAF therapy. Future research should include more high-quality RCTs to further validate these findings.

## Data Availability

The original contributions presented in the study are included in the article/Supplementary Material, further inquiries can be directed to the corresponding author.
