# Supplementary material for: Metabolic effects and cardiovascular disease risks of TDF or TAF in patients with chronic hepatitis B: a systematic review and meta-analysis
Source: Front Pharmacol. 2025 Jun 30;16:1604972. doi: 10.3389/fphar.2025.1604972 (PMC12256854; doi:10.3389/fphar.2025.1604972)
Supplement: Supplementary file 1 [file DataSheet1.docx]

Metabolic Effects and Cardiovascular Risks of TDF or TAF in Chronic Hepatitis B Patients: A Systematic Review and Meta-Analysis

Yuan-Hai Zhou^1,2^, Nan Cai^2,3^, Yu-Xin Chen^2,3^, Yong-Lu Su^2,3^, Peng Hu^1,2,4^

Supplementary Table 1. Search Strategy

| **P** | subject terms | free words |
| --- | --- | --- |
|  | Hepatitis B, chronic | Hepatitis B Virus Infection, Chronic  Chronic Hepatitis B Virus Infection  Chronic HBV  CHB  Chronic type B hepatitis  Chronic hepatitis type B  Chronic Hepatitis B |

| I | subject terms | free words |
| --- | --- | --- |
|  | [Tenofovir](https://www.ncbi.nlm.nih.gov/mesh/2009864) | Tenofovir Disoproxil Fumarate  Disoproxil Fumarate, Tenofovir  Fumarate, Tenofovir Disoproxil  Viread  Tenofovir Disoproxil  Disoproxil, Tenofovir  tenofovir disoproxil fumarate  TDF  Tenofovir alafenamide  Tenofovir-A  Vemlidy  TAF |

| O | subject terms | free words |
| --- | --- | --- |
|  | Cardiovascular Disease  Dyslipidemias  Hyperlipidemias  Hypercholesterolemia  Cholesterol  Cholesterol, HDL  Lipoproteins, HDL  Cholesterol, LDL  Lipoproteins, LDL  Triglycerides | Cardiac Events  Adverse Cardiac Event  Major Cardiovascular adverse events  MACE  Major Adverse Cardiac Events  Coronary Artery Disease  Coronary Atherosclerosis  Dyslipidemia  Dyslipoproteinemia  Hyperlipidemia  Hyperlipemia  Lipidemia  Lipemia  Hypercholesterolemia  Hypercholesteremia  Cholesterol  HDL  Low-density lipoprotein  LDL  Triglyceride  Triacylglycerol  Triacylglyceride  Low lipid |

| **Databases** | **Search Strategy** |
| --- | --- |
| **PubMed** | Search: (((((((((Hepatitis B, chronic[MeSH Terms]) OR (Hepatitis B Virus Infection, Chronic[Title/Abstract])) OR (Chronic Hepatitis B Virus Infection[Title/Abstract])) OR (Chronic HBV[Title/Abstract])) OR (CHB[Title/Abstract])) OR (Chronic type B hepatitis[Title/Abstract])) OR (Chroinic hepatitis type B[Title/Abstract])) OR (Chronic Hepatitis B[Title/Abstract])) AND (((((((((((((Tenofovir[MeSH Terms]) OR (Tenofovir Disoproxil Fumarate[Title/Abstract])) OR (Disoproxil Fumarate, Tenofovir[Title/Abstract])) OR (Fumarate, Tenofovir Disoproxil[Title/Abstract])) OR (Viread[Title/Abstract])) OR (Tenofovir Disoproxil[Title/Abstract])) OR (Disoproxil, Tenofovir[Title/Abstract])) OR (tenofovir disoproxil fumarate[Title/Abstract])) OR (TDF[Title/Abstract])) OR (Tenofovir alafenamide[Title/Abstract])) OR (Tenofovir-A[Title/Abstract])) OR (Vemlidy[Title/Abstract])) OR (TAF[Title/Abstract]))) AND (((((((((((((((((((((((((((((((((Cardiovascular Disease[MeSH Terms]) OR (Dyslipidemias[MeSH Terms])) OR (Hyperlipidemias[MeSH Terms])) OR (Hypercholesterolemia[MeSH Terms])) OR (Cholesterol[MeSH Terms])) OR (Cholesterol, HDL[MeSH Terms])) OR (Lipoproteins, HDL[MeSH Terms])) OR (Cholesterol, LDL[MeSH Terms])) OR (Lipoproteins, LDL[MeSH Terms])) OR (Triglycerides[MeSH Terms])) OR (Cardiac Events[Title/Abstract])) OR (Adverse Cardiac Event[Title/Abstract])) OR (Major Cardiovascular adverse events[Title/Abstract])) OR (MACE[Title/Abstract])) OR (Major Adverse Cardiac Events[Title/Abstract])) OR (Coronary Artery Disease[Title/Abstract])) OR (Coronary Atheroscleroses[Title/Abstract])) OR (Dyslipidemia[Title/Abstract])) OR (Dyslipoproteinemia[Title/Abstract])) OR (Hyperlipidemia[Title/Abstract])) OR (Hyperlipemia[Title/Abstract])) OR (Lipidemia[Title/Abstract])) OR (Lipemia[Title/Abstract])) OR (Hypercholesterolemia[Title/Abstract])) OR (Hypercholesteremia[Title/Abstract])) OR (Cholesterol[Title/Abstract])) OR (HDL[Title/Abstract])) OR (Low-density lipoprotein[Title/Abstract])) OR (LDL[Title/Abstract])) OR (Triglyceride[Title/Abstract])) OR (Triacylglycerol[Title/Abstract])) OR (Triacylglyceride[Title/Abstract])) OR (Low lipid[Title/Abstract])) |
| **EMBASE** | 1. 'hepatitis b, chronic':ab,ti OR 'hepatitis b virus infection, chronic':ab,ti OR 'chronic hepatitis b virus infection':ab,ti OR 'chronic hbv':ab,ti OR 'chb':ab,ti OR 'chronic type b hepatitis':ab,ti OR 'chroinic hepatitis type b':ab,ti OR 'chronic hepatitis b':ab,ti 2. 'tenofovir':ab,ti OR 'disoproxil fumarate, tenofovir':ab,ti OR 'fumarate, tenofovir disoproxil':ab,ti OR 'viread':ab,ti OR 'tenofovir disoproxil':ab,ti OR 'disoproxil, tenofovir':ab,ti OR 'tenofovir disoproxil fumarate':ab,ti OR 'tdf':ab,ti OR 'tenofovir alafenamide':ab,ti OR 'tenofovir-a':ab,ti OR 'vemlidy':ab,ti OR 'taf':ab,ti 3. 'cardiovascular disease':ab,ti OR 'dyslipidemias':ab,ti OR 'hyperlipidemias':ab,ti OR 'cholesterol, hdl':ab,ti OR 'lipoproteins, hdl':ab,ti OR 'cholesterol, ldl':ab,ti OR 'lipoproteins, ldl':ab,ti OR 'triglycerides':ab,ti OR 'cardiac events':ab,ti OR 'adverse cardiac event':ab,ti OR 'major cardiovascular adverse events':ab,ti OR 'mace':ab,ti OR 'major adverse cardiac events':ab,ti OR 'coronary artery disease':ab,ti OR 'coronary atheroscleroses':ab,ti OR 'dyslipidemia':ab,ti OR 'dyslipoproteinemia':ab,ti OR 'hyperlipidemia':ab,ti OR 'hyperlipemia':ab,ti OR 'lipidemia':ab,ti OR 'lipemia':ab,ti OR 'hypercholesterolemia':ab,ti OR 'hypercholesteremia':ab,ti OR 'cholesterol':ab,ti OR 'hdl':ab,ti OR 'low-density lipoprotein':ab,ti OR 'ldl':ab,ti OR 'triglyceride':ab,ti OR 'triacylglycerol':ab,ti OR 'triacylglyceride':ab,ti OR 'low lipid':ab,ti 4. #1 AND #2 AND #3 |
| **Cochrane** | 1. (Hepatitis B, chronic):ti,ab,kw OR (Hepatitis B Virus Infection, Chronic):ti,ab,kw OR (Chronic Hepatitis B Virus Infection):ti,ab,kw OR (Chronic HBV):ti,ab,kw OR (CHB):ti,ab,kw OR (Chronic type B hepatitis):ti,ab,kw OR (Chroinic hepatitis type B):ti,ab,kw OR (Chronic Hepatitis B):ti,ab,kw 2. (Tenofovir):ti,ab,kw OR (Tenofovir Disoproxil Fumarate):ti,ab,kw OR (Disoproxil Fumarate, Tenofovir):ti,ab,kw OR (Fumarate, Tenofovir Disoproxil):ti,ab,kw OR (Viread):ti,ab,kw OR (Tenofovir Disoproxil):ti,ab,kw OR (Disoproxil, Tenofovir):ti,ab,kw OR (tenofovir disoproxil fumarate):ti,ab,kw OR (TDF):ti,ab,kw OR (Tenofovir alafenamide):ti,ab,kw OR (Tenofovir-A):ti,ab,kw OR (Vemlidy):ti,ab,kw OR (TAF):ti,ab,kw 3. (Cardiovascular Disease):ti,ab,kw OR (Dyslipidemias):ti,ab,kw OR (Hyperlipidemias):ti,ab,kw OR (Hypercholesterolemia):ti,ab,kw OR (Cholesterol):ti,ab,kw OR (Cholesterol, HDL):ti,ab,kw OR (Lipoproteins, HDL):ti,ab,kw OR (Cholesterol, LDL):ti,ab,kw OR (Lipoproteins, LDL):ti,ab,kw OR (Triglycerides):ti,ab,kw OR (Cardiac Events):ti,ab,kw OR (Adverse Cardiac Event):ti,ab,kw OR (Major Cardiovascular adverse events):ti,ab,kw OR (MACE):ti,ab,kw OR (Major Adverse Cardiac Events):ti,ab,kw OR (Coronary Artery Disease):ti,ab,kw OR (Coronary Atheroscleroses):ti,ab,kw OR (Dyslipidemia):ti,ab,kw OR (Dyslipoproteinemia):ti,ab,kw OR (Hyperlipidemia):ti,ab,kw OR (Hyperlipemia):ti,ab,kw OR (Lipidemia):ti,ab,kw OR (Lipemia):ti,ab,kw OR (Hypercholesterolemia):ti,ab,kw OR (Hypercholesteremia):ti,ab,kw OR (Cholesterol):ti,ab,kw OR (HDL):ti,ab,kw OR (Low-density lipoprotein):ti,ab,kw OR (LDL):ti,ab,kw OR (Triglyceride):ti,ab,kw OR (Triacylglycerol):ti,ab,kw OR (Triacylglyceride):ti,ab,kw OR (Low lipid):ti,ab,kw 4. #1 AND #2 AND #3 |
| **Web of Science** | 1. TS=（"Hepatitis B, chronic" OR "Hepatitis B Virus Infection, Chronic" OR "Chronic Hepatitis B Virus Infection" OR "Chronic HBV" OR "CHB" OR "Chronic type B hepatitis" OR "Chroinic hepatitis type B" OR "Chronic Hepatitis B"） 2. TS=（"Tenofovir" OR "Tenofovir Disoproxil Fumarate" OR "Disoproxil Fumarate, Tenofovir" OR "Fumarate, Tenofovir Disoproxil" OR "Viread" OR "Tenofovir Disoproxil" OR "Disoproxil, Tenofovir" OR "tenofovir disoproxil fumarate" OR "TDF" OR "Tenofovir alafenamide" OR "Tenofovir-A" OR "Vemlidy" OR "TAF"） 3. TS=("Cardiovascular Disease" OR "Dyslipidemias" OR "Hyperlipidemias" OR "Hypercholesterolemia" OR "Cholesterol" OR "Cholesterol, HDL" OR "Lipoproteins, HDL" OR "Cholesterol, LDL" OR "Lipoproteins, LDL" OR "Triglycerides" OR "Cardiac Events" OR "Adverse Cardiac Event" OR "Major Cardiovascular adverse events" OR "MACE" OR "Major Adverse Cardiac Events" OR "Coronary Artery Disease" OR "Coronary Atheroscleroses" OR "Dyslipidemia" OR "Dyslipoproteinemia" OR "Hyperlipidemia" OR "Hyperlipemia" OR "Lipidemia" OR "Lipemia" OR "Hypercholesterolemia" OR "Hypercholesteremia" OR "Cholesterol" OR "HDL" OR "Low-density lipoprotein" OR "LDL" OR "Triglyceride" OR "Triacylglycerol" OR "Triacylglyceride" OR "Low lipid") 4. #1 AND #2 AND #3 |

Supplementary Table 2. Search Results

| **No.** | **DB** | **Results** | **Duplication** | **without duplication** |
| --- | --- | --- | --- | --- |
| 1 | PubMed (Medline) | 50 | 122 | 142 |
| 2 | EMBASE | 124 |  |  |
| 3 | Cochrane Library | 37 |  |  |
| 4 | Web of Science | 53 |  |  |
| 5 | Repeat the results | 264 |  |  |

Supplementary Table 3. Newcastle-Ottawa Quality Assessment Form for Cohort Studies for Assessing the Quality of All Eligible Nonrandomized Comparative Studies

| Study | Selection | Comparability | Outcome | Total score |
| --- | --- | --- | --- | --- |
| Pin-Nan Cheng et al,2024 | 4 | 2 | 2 | 8 |
| Hyunjae Shin et al,2024 | 4 | 2 | 2 | 8 |
| Hyeyeon Hong,2024 | 3 | 2 | 2 | 7 |
| Wenjuan Zhao et al,2024 | 3 | 2 | 3 | 8 |
| Rui-Min Lai et al,2023 | 3 | 2 | 2 | 7 |
| Jing Wen Chen et al,2022 | 4 | 2 | 2 | 8 |
| Eiichi Ogawa et al,2022 | 4 | 2 | 2 | 8 |
| Jihye Lim et al,2022 | 4 | 2 | 3 | 9 |
| Rahmet Guner et al,2022 | 4 | 2 | 3 | 9 |
| Yeqiong Y. Zhang et al,2022 | 4 | 1 | 2 | 7 |
| Kazuharu Suzuki et al,2022 | 4 | 2 | 2 | 8 |
| Omer Karasahin et al,2022 | 4 | 2 | 3 | 9 |
| Qi Zhang et al,2022 | 3 | 2 | 2 | 7 |
| Ming-Lun Yeh et al,2022 | 3 | 2 | 2 | 7 |
| Joonho Jeong et al,2021 | 4 | 2 | 3 | 9 |
| Irem Akdemir Kalkan et al,2021 | 4 | 2 | 2 | 8 |


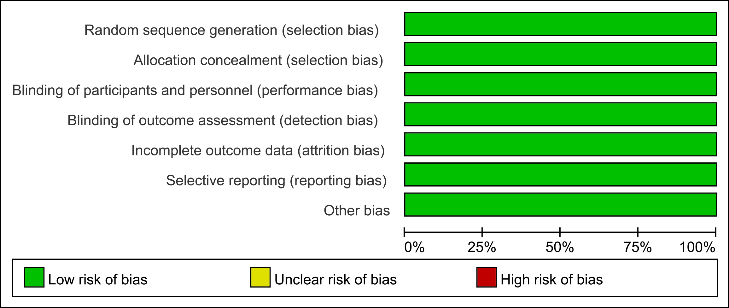

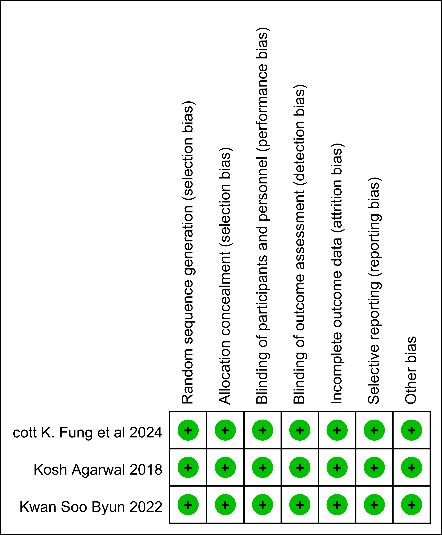


Supplementary Fig.1 Quality assessment of randomized control trials

Supplementary Table4. Changes in lipid **Levels** at 24, 48, 72 and 96 weeks of CHB treated with TAF

|  | Forest plots | Funnel Plots | Influence analysis |
| --- | --- | --- | --- |
| TG-24w | 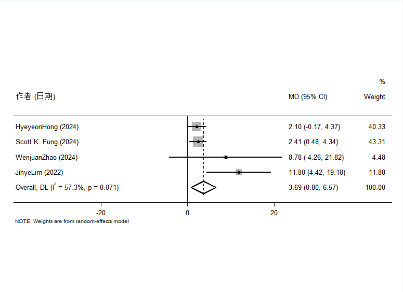 | 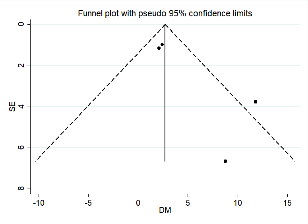 | 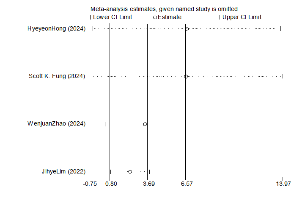 |
| TG-48w | 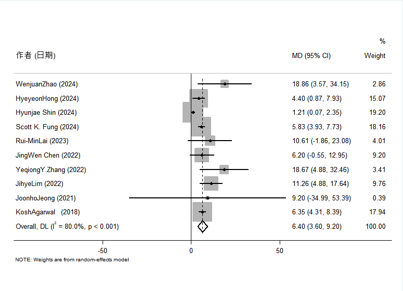 | 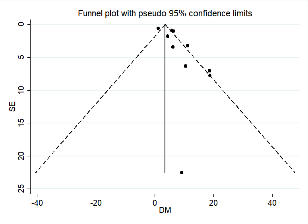 | 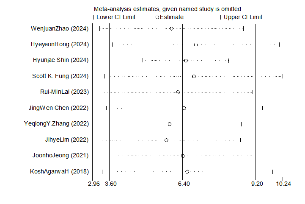 |
| TG-72w | 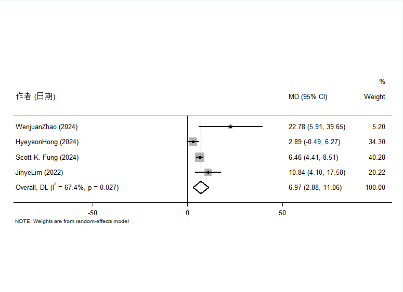 | 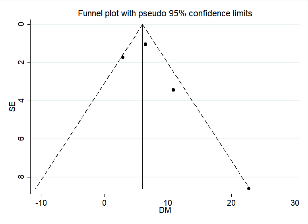 | 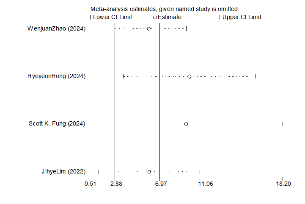 |
| TG-96w | 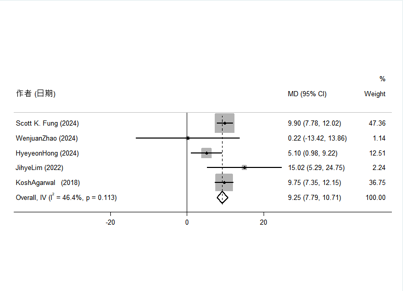 | 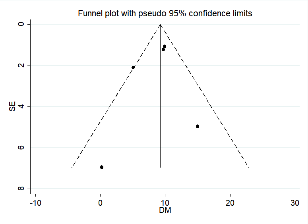 | 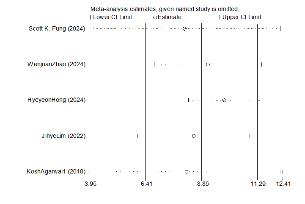 |
| TC-24w | 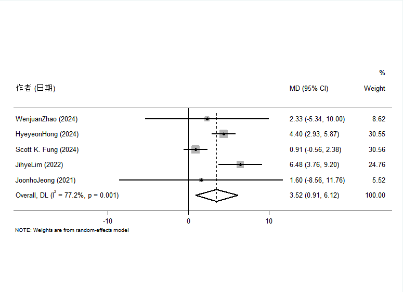 | 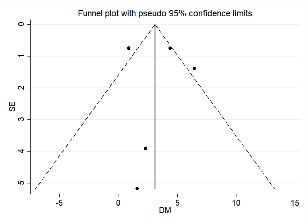 | 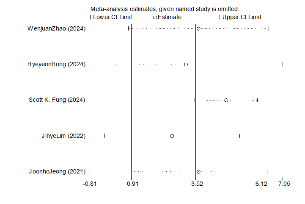 |
| TC-48w | 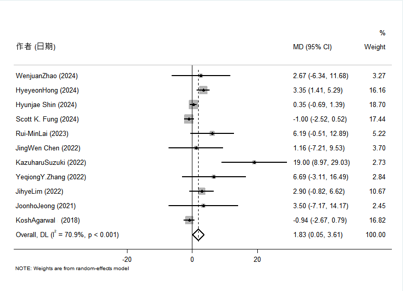 | 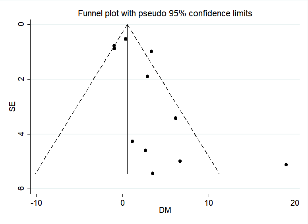 | 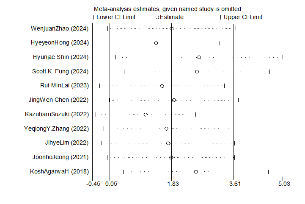 |
| TC-72w | 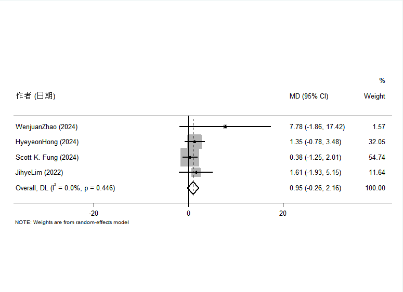 | 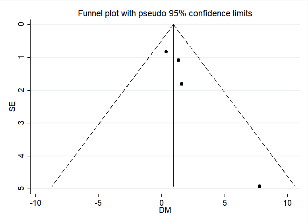 | 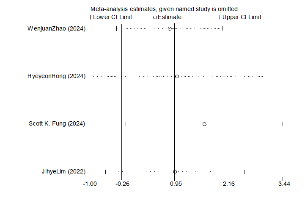 |
| TC-96w | 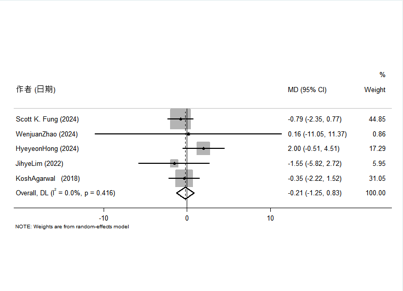 | 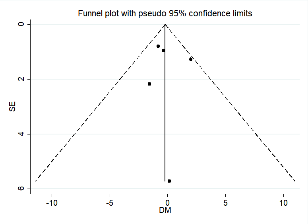 | 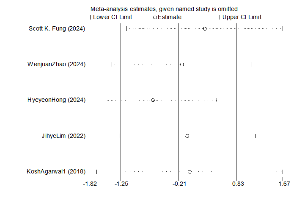 |
| HDL-24w | 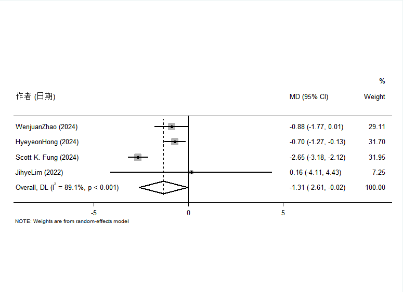 | 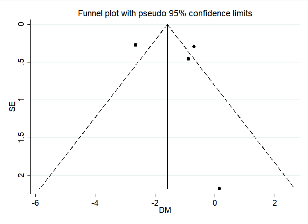 | 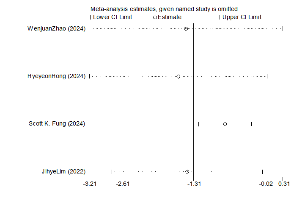 |
| HDL-48w | 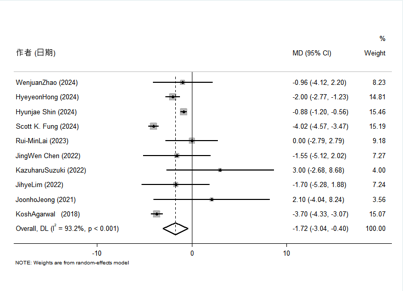 | 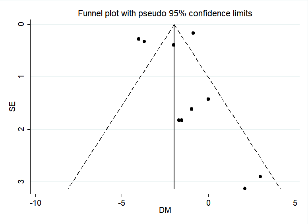 | 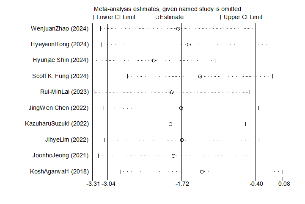 |
| HDL-72w | 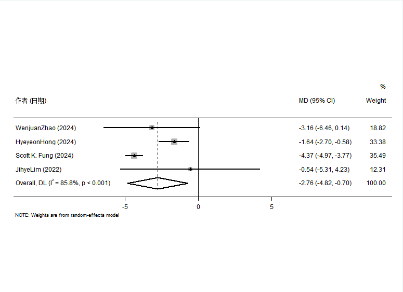 | 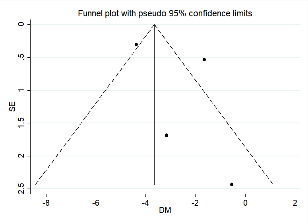 | 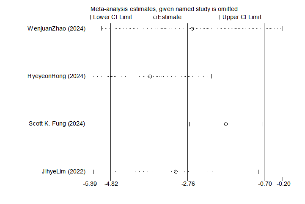 |
| HDL-96w | 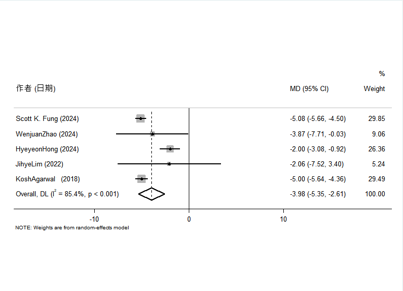 | 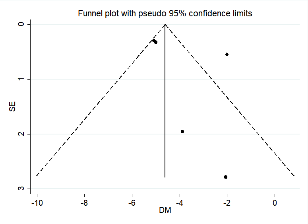 | 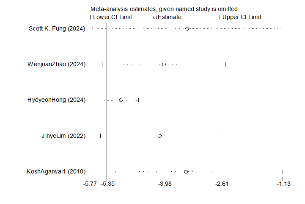 |
| LDL-24w | 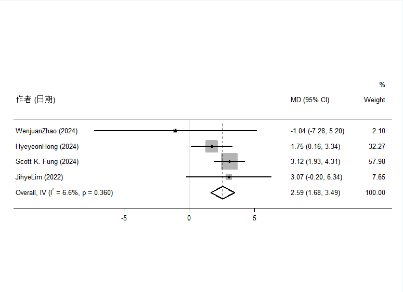 | 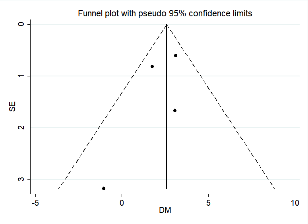 | 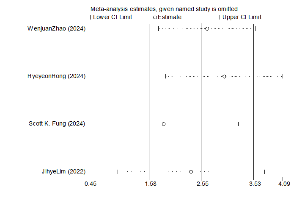 |
| LDL-48w | 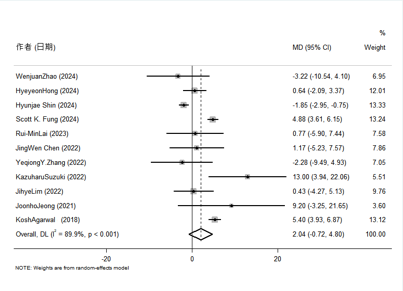 | 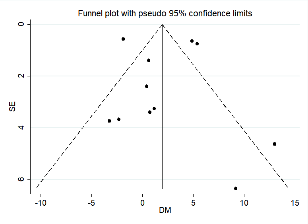 | 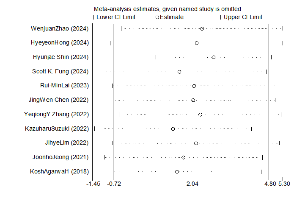 |
| LDL-72w | 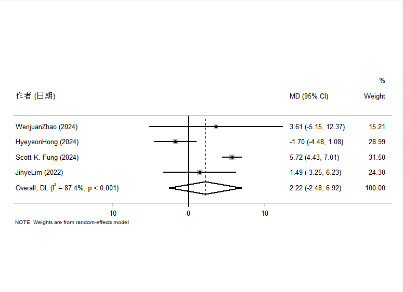 | 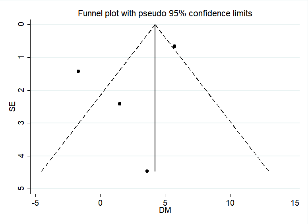 | 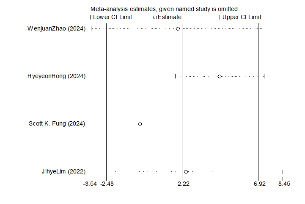 |
| LDL-96w | 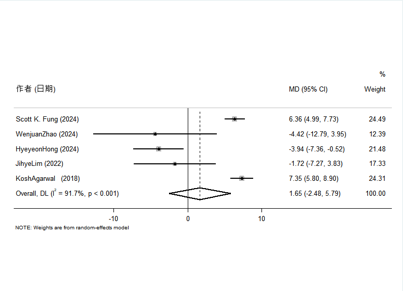 | 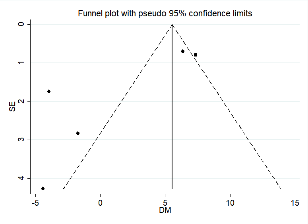 | 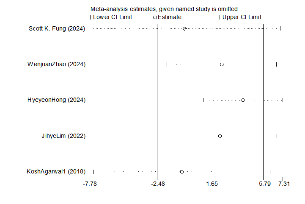 |
| TC/HDL-24W | 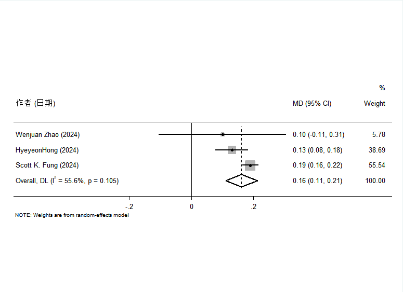 | 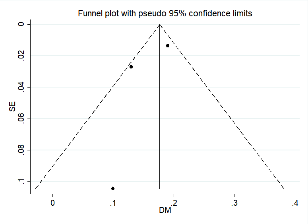 | 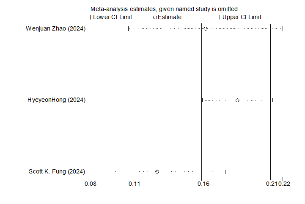 |
| TC/HDL-48W | 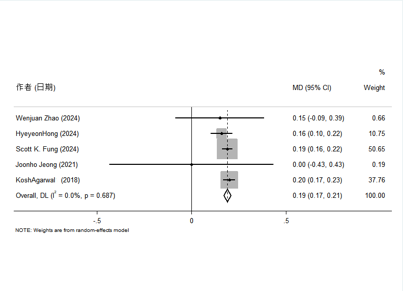 | 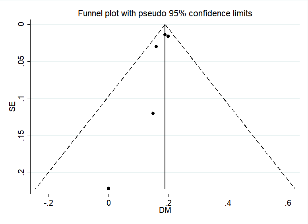 | 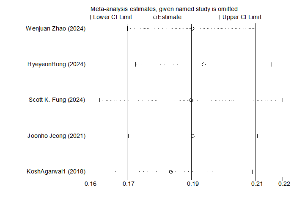 |
| TC/HDL-72W | 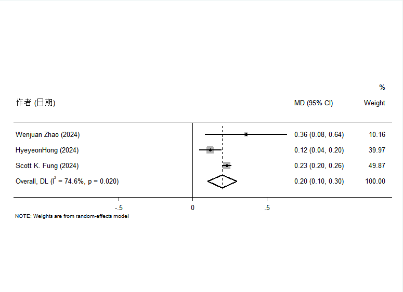 | 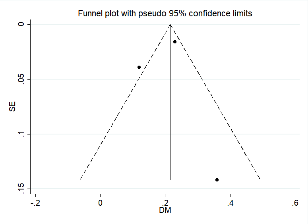 | 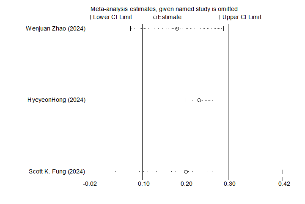 |
| TC/HDL-96W | 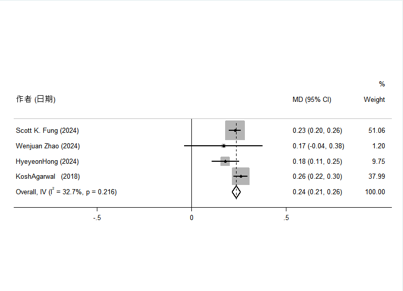 | 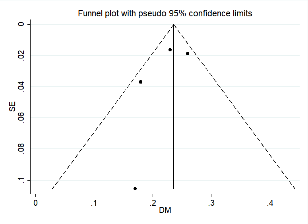 | 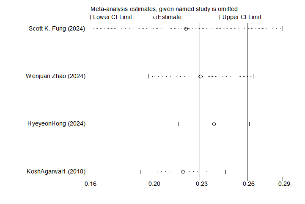 |

Supplementary Table5. Changes in lipid **Levels** at 24, 48, 72 and 96 weeks of CHB treated with TDF

|  | Forest plots` | Funnel Plots | Influence analysis |
| --- | --- | --- | --- |
| TG-24w | 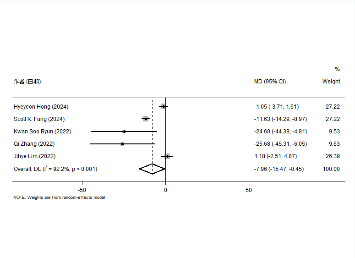 | 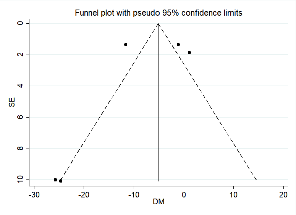 | 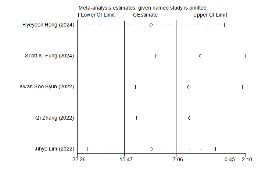 |
| TG -48w | 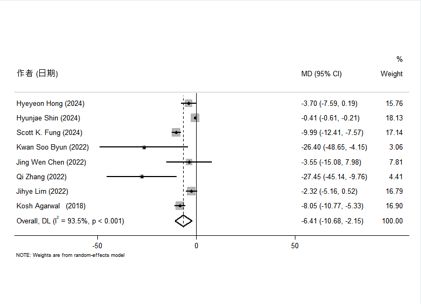 | 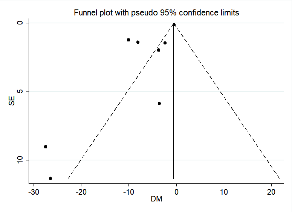 | 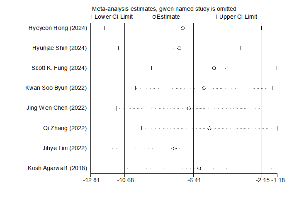 |
| TG -72w | 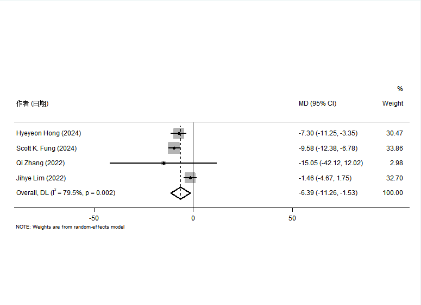 | 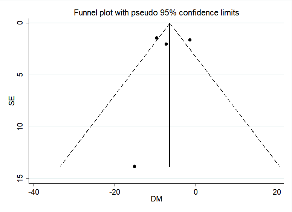 | 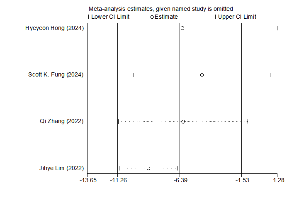 |
| TG -96w | 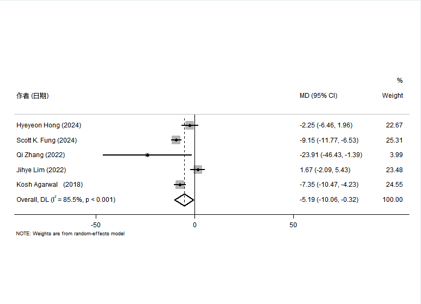 | 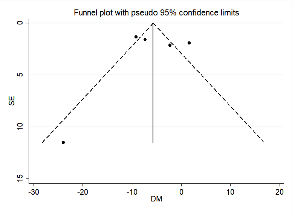 | 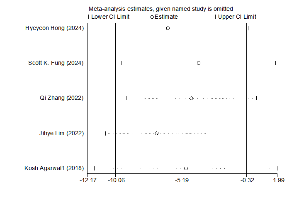 |
| TC-24w | 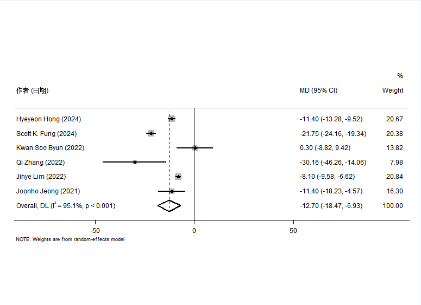 | 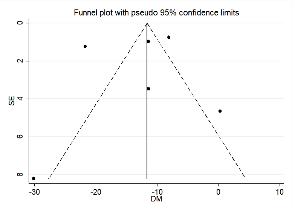 | 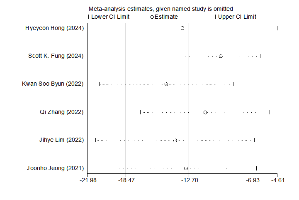 |
| TC -48w | 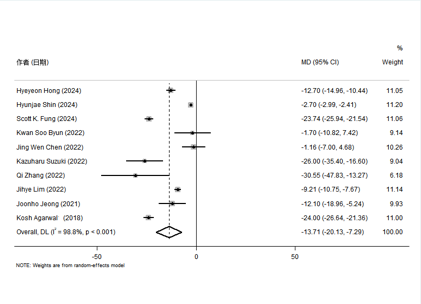 | 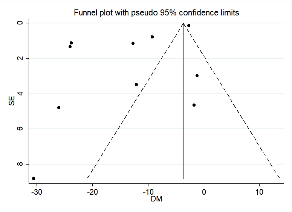 | 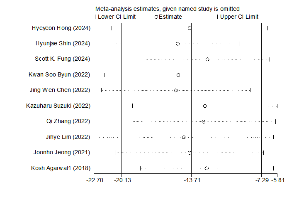 |
| TC -72w | 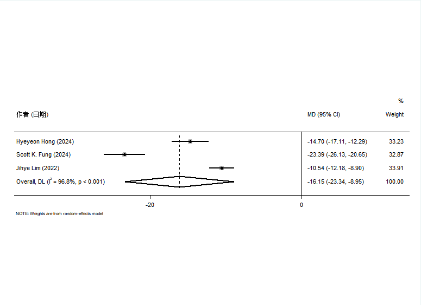 | 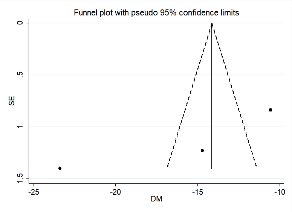 | 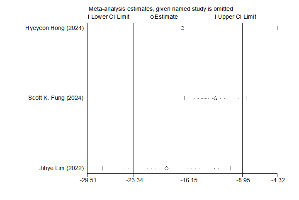 |
| TC -96w | 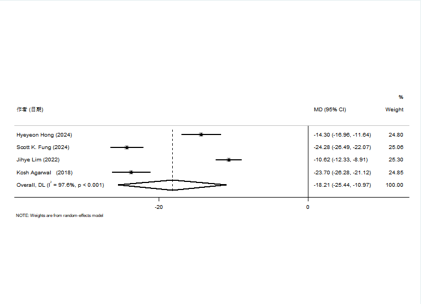 | 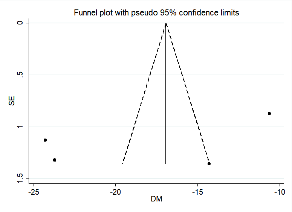 | 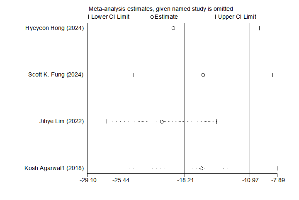 |
| HDL-24w | 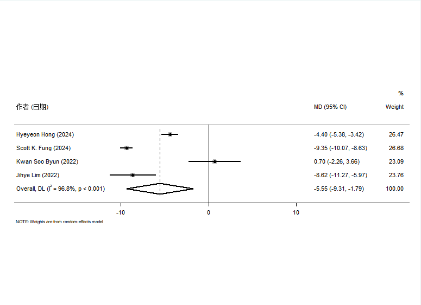 | 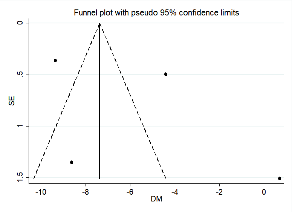 | 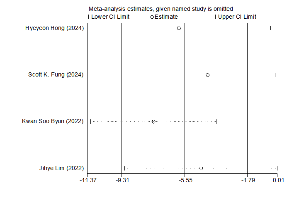 |
| HDL-48w | 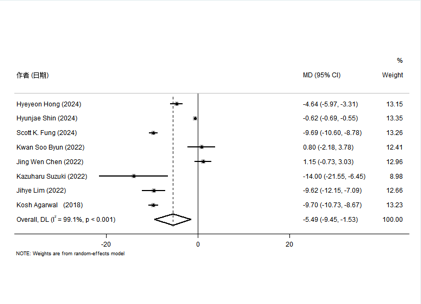 | 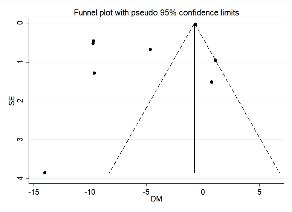 | 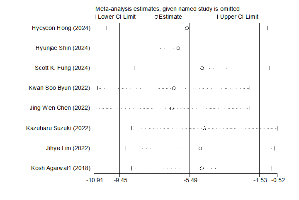 |
| HDL-72w | 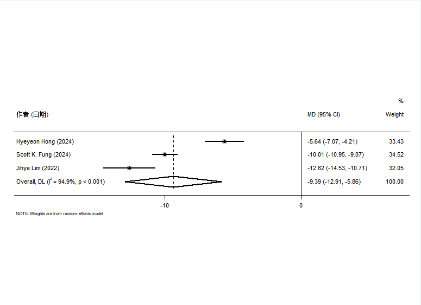 | 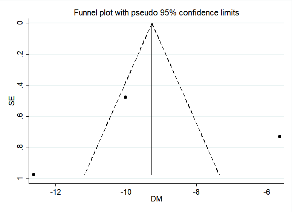 | 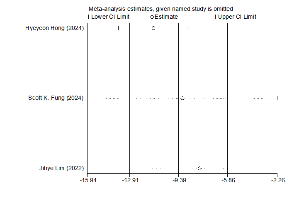 |
| HDL-96w | 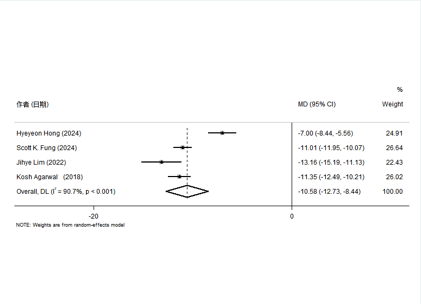 | 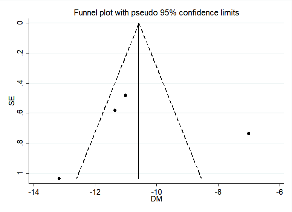 | 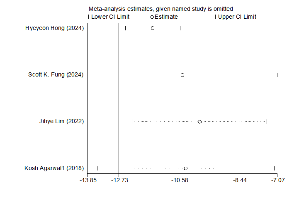 |
| LDL-24w | 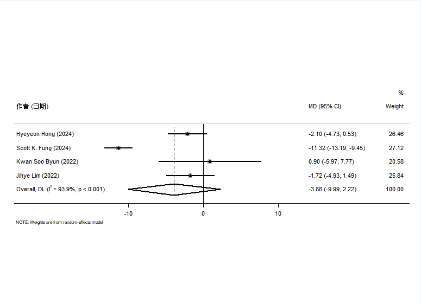 | 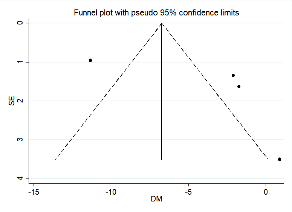 |  |
| LDL-48w |  |  |  |
| LDL-72w |  |  |  |
| LDL-96w |  |  |  |
| TC/HDL-24W |  |  |  |
| TC/HDL-48W |  |  |  |
| TC/HDL-72W |  |  |  |
| TC/HDL-96W |  |  |  |

Supplementary Table6. Changes in 10-year cardiovascular risk following TAF and TDF treatment

|  | Forest plots` | Funnel Plots | Influence analysis |
| --- | --- | --- | --- |
| TAF |  |  |  |
| TDF |  |  |  |

Supplementary Table7. Changes in body weight and blood glucose at 48 weeks of TAF treatment

|  | Forest plots` | Funnel Plots | Influence analysis |
| --- | --- | --- | --- |
| body weight |  |  |  |
| blood glucose |  |  |  |

Supplementary Table8. Changes in body weight and blood glucose at 48 weeks of TDF treatment

|  | Forest plots` | Funnel Plots | Influence analysis |
| --- | --- | --- | --- |
| body weight |  |  |  |
| blood glucose |  |  |  |

Supplementary Table9. Changes in lipid **Levels** at 24weeks of switched to TAF treatment

|  | Forest plots` | Funnel Plots | Influence analysis |
| --- | --- | --- | --- |
| TG |  |  |  |
| TC |  |  |  |
| HDL |  |  |  |
| LDL |  |  |  |
| TC/HDL |  |  |  |
| BW |  |  |  |

Supplementary Table10. Subgroup Analysis of TDF/TAF Monotherapy by Study Design

|  | Non-randomized Studies | Randomized Controlled Trials |
| --- | --- | --- |
| **TAF monotherapy for 48 weeks** | |  |
| TG |  |  |
| TC |  |  |
| HDL |  |  |
| LDL |  |  |
| **TDF monotherapy for 48 weeks** | |  |
| TG |  |  |
| TC |  |  |
| HDL |  |  |
| LDL |  |  |

Supplementary Table11. Subgroup Analysis of Switch from TDF to TAF Monotherapy by Study Design

| **Switch from TDF to TAF for 24 weeks (Non-randomized Studies)** | |
| --- | --- |
| TG |  |
| TC |  |
| HDL |  |
| LDL |  |

Supplementary Table12. Impact of Study Design (RCTs vs. Observational Studies) on Metabolic Outcomes

|  | Non-randomized Studies | | | |  | Randomized Controlled Trials | | | |
| --- | --- | --- | --- | --- | --- | --- | --- | --- | --- |
|  | N | MD (95% CI) | I2 | p-value |  | N | MD (95% CI) | I2 | p-value |
| **TAF monotherapy for 48 weeks** | | | | |  |  |  |  |  |
| TG ((mg/dL) | 8 | 7.39(3.12, 11.65) | 72.40% | 0.001 |  | 2 | 6.07(4.68, 7.46) | 0.00% | 0.715 |
| TC (mg/dL) | 9 | 3.57(1.07, 6.06) | 66.10% | 0.003 |  | 2 | -0.97(-2.11, 0.17) | 0.00% | 0.959 |
| HDL (mg/dL) | 8 | -1.03(-1.32, -0.73) | 33.60% | 0.16 |  | 2 | -3.88(-4.30, -3.47) | 0.00% | 0.45 |
| LDL (mg/dL) | 9 | -1.14(-2.09, -0.19) | 52.60% | 0.03 |  | 2 | 5.10(4.14, 6.06) | 0.00% | 0.599 |
| **TDF monotherapy for 48 weeks** | | | | |  |  |  |  |  |
| TG ((mg/dL) | 5 | -2.68(-5.75, -1.38) | 70.80% | 0.008 |  | 3 | -9.33(-12.10, -6.56) | 41.00% | 0.18 |
| TC (mg/dL) | 7 | -10.90(-15.83, -5.98) | 96.60% | 0.001 |  | 3 | -18.8(-25.39, -12.21) | 90.90% | 0.001 |
| HDL (mg/dL) | 5 | -4.45(-7.77, -1.12) | 96.00% | 0.001 |  | 3 | -6.64(-10.23, -3.04) | 95.60% | 0.001 |
| LDL (mg/dL) | 5 | -1.16(-1.32, -0.99) | 0.00% | 0.48 |  | 3 | -8.54(-12.35, -4.73) | 80.80% | 0.005 |
| **Switch from TDF to TAF for 24 weeks** | | | | |  |  |  |  |  |
| TG ((mg/dL) | 4 | 9.31(2.21, 16.41) | 61.20% | 0.052 |  |  |  |  |  |
| TC (mg/dL) | 5 | 16.55(8.91, 24.20) | 85.50% | 0.001 |  |  |  |  |  |
| HDL (mg/dL) | 5 | 3.89(1.47, 6.30) | 75.00% | 0.003 |  |  |  |  |  |
| LDL (mg/dL) | 5 | 11.22(7.38, 15.05) | 67.20% | 0.016 |  |  |  |  |  |
